# The role of vitamin D receptor gene polymorphisms in gestational diabetes mellitus susceptibility: a meta-analysis

**DOI:** 10.1186/s13098-021-00764-y

**Published:** 2021-12-13

**Authors:** Sai Liu

**Affiliations:** Department of Emergency, Beijing Luhe Hospital, Capital Medical University, No. 82, Xinhua south road, Tongzhou District, Beijing, 101149 China

**Keywords:** VDR, GDM, Vitamin D receptor, Gestational diabetes mellitus, Meta-analysis

## Abstract

**Background:**

Gestational diabetes mellitus (GDM) is a common disease during pregnancy. The association of vitamin D receptor (VDR) polymorphisms with GDM is still controversial. This study aimed to assess the associations between VDR polymorphisms and GDM risk.

**Methods:**

We searched Cochrane Library, PubMed, and Embase electronic database for all eligible studies published from Jan 1, 1980 to December 31, 2020 to conduct a Meta-analysis. We analyzed four VDR polymorphisms: BsmI (rs1544410), ApaI (rs7975232), TaqI (rs731236), and FokI (rs2228570). Inclusion Criteria: (1) The data can be evaluated; (2) case–control study; and (3) meeting the Hardy–Weinberg’s law. Exclusion criteria: (1) Insufficient or extractable data; (2) Severe publication bias in the data; and (3) duplicate publications. We eventually included 15 studies in seven articles, including 2207 cases and 2706 controls.

**Results:**

We eventually included 15 studies in seven articles, including 2207 cases and 2706 controls. The data showed that ApaI (rs7975232) VDR gene polymorphism was related with the risk of GDM for the comparison of CC vs AA and recessive model in overall population and FokI (rs2228570) VDR gene polymorphism was associated with the risk of GDM for recessive model in overall population. BsmI (rs1544410) polymorphism was not related with the risk of GDM in overall population. However, in the analysis of subgroups grouped by race, BsmI (rs1544410) has certain correlations. And, the data suggested the TaqI (rs731236) polymorphism was not associated with GDM.

**Conclusion:**

Based on the meta-analysis, VDR ApaI (rs7975232) and FokI (rs2228570) polymorphisms increase susceptibility to GDM. In the future, it can be used to diagnose and screen molecular biomarkers for GDM patients.

## Background

Gestational diabetes mellitus (GDM) is defined as glucose intolerance diagnosed during pregnancy [1]. GDM is characterized by increased insulin resistance, hyperglycemia, and obesity [2–4]. The prevalence of GDM is increasing in decades and floating from 1.7 to 11.6% among populations [5]. Although considerable research effort has been focused on GDM, the pathophysiology of the disease remains incompletely understood. Genetic and environmental factors play an important role in the etiology of GDM [2].

Vitamin D deficiency is associated with diabetes mellitus [6–8]. Vitamin D receptor (VDR) gene polymorphisms may contribute to development of diabetes mellitus through calcium metabolism alteration and modulation of insulin secretion [9–11]. Three single nucleotide polymorphisms BsmI, ApaI and TaqI of the VDR gene were found in the major untranslated regions that regulate gene expression. FokI is a T > C substitution that results in exon 2 [12, 13]. The above four VDR gene polymorphisms all have a certain effect on insulin production, and secretion plays a role in the pathogenesis of GDM. Therefore, VDR gene polymorphisms may plays a role in the pathogenesis of GDM.

Many studies have researched the role of VDR gene polymorphisms in GDM. It is reported that VDR has four well-characterized di-allelic polymorphisms: BsmI (A > G, rs1544410), ApaI (A > C, rs7975232), TaqI (T > C, rs731236), and FokI (C > T, rs2228570). However, the results of these studies are still uncertain [13–19]. Different research teams and research designs might lead to differences in results. The objective is to clarify the effect of VDR gene polymorphisms on GDM risk, we conducted a meta-analysis of all eligible case–control studies.

## Methods

### Search strategy

We identified the keywords “VDR” OR “vitamin D receptor” AND “polymorphism” OR “variant” OR “allele” OR “genotype” OR “gestational diabetes” OR “gestational diabetes mellitus” OR “GDM” to search the articles in Cochrane Library, PubMed, and Embase electronic database. All articles published until December 31, 2020. In addition, manually search the article's reference list for more literature. This article does not collect unpublished data. When multiple articles contain studies of the same population, complete studies were chosen in this study. The language of the publication is limited to English or at least an English abstract.

### Inclusion and exclusion criteria

Inclusion Criteria: (1) The data can be evaluated; (2) case–control study; and (3) meeting the Hardy–Weinberg’s law. Exclusion criteria: (1) Insufficient or extractable data; (2) Severe publication bias in the data; and (3) duplicate publications.

### Data extraction

The data was independently evaluated by two reviewers according to include and exclude criteria for these documents, discuss whether can be included in the meta-analysis. The difference was not resolved until the consensus of each item was reached. The following information was recorded for each study: author’s name, year of publication, country of origin, racial descent, source of the control population, genotyping methods, matched factors as well as adjusted factors, number of cases and controls.

### Statistical analysis

ORs (odds ratios) and 95% CIs were used to estimate the relationships between VDR gene polymorphism and GDM. For heterogeneity detection, we chose the P value to measure. If P < 0.05, we chose the random effect model, otherwise chose the fixed effect model. For publication bias we calculated Egger and Begg’ test, respectively (P < 0.05 was considered representative of statistically significant publication bias). If P < 0.05, it was considered biased. Hardy–Weinberg’s law was detected in all control groups. This meta-analysis was performed using STATA (version 14.0; US).

## Results

### Study selection

**Fig. 1:**
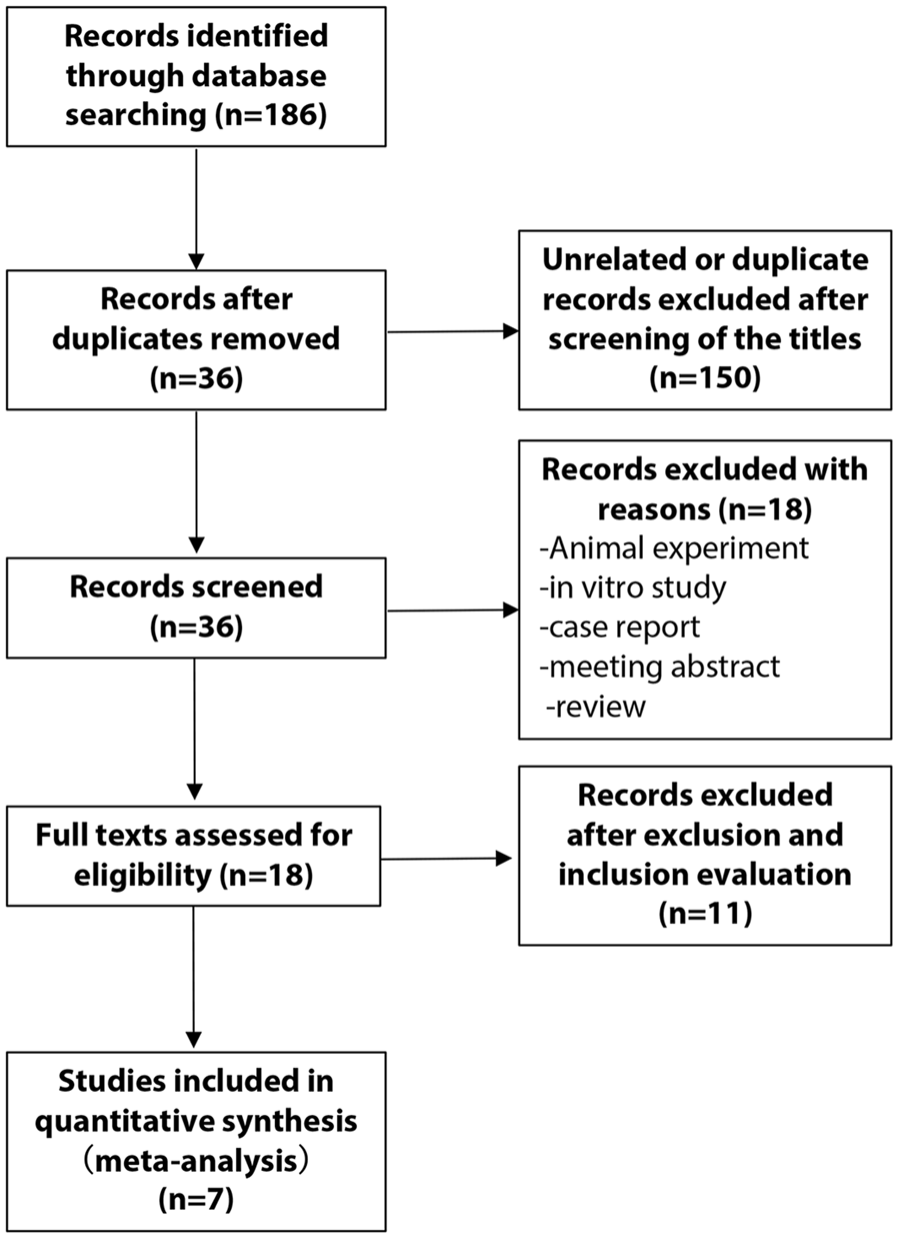
Flow diagram detailing procedures of selecting eligible studies

We found 186 records through a full search of the database. After several rounds of screening, 36 articles met our requirements. After two individuals independently evaluated the inclusion and exclusion criteria, 15 case–control studies in a total of seven articles were included in the study [13–19]. We identified 186 articles from the database, and after excluding irrelevant and duplicate research, 36 articles entered the next step of analysis. According to the inclusion and exclusion criteria, seven articles were included in our study. The specific retrieval process was shown in Fig. 1.

### Study characteristics

We identified 15 independent studies in seven eligible reports, including 2207 cases and 2706 controls. The main characteristics of all the studies included in our study were shown in Table 1. There were 5 case–control studies on BsmI (rs1544410) [14–17, 19], 4 case–control studies on TaqI (rs731236) [13–16], 3 case–control studies on FokI (rs2228570) [14, 15, 17] and 3 case–control studies on ApaI (rs7975232) [13–15]. 15 independent studies consisted of 4 Asian [16, 19], 3 African [17] and 8 Caucasian populations [13–15, 18].

**Table 1:** Basic information of the original articles included in this meta-analysis

Site	First author	Ethnicity	Year	Design	Methods	Case			Control		
BsmI (rs1544410)						GG	AA	GA	GG	AA	GA
	Qi Juan	Asian	2013	HBC	PCR–RFLP	0	58	22	0	70	10
	Hesham A	African	2015	PBC	PCR–RFLP	11	61	40	66	40	112
	Mahmut Apaydın	Caucasian	2019	HBC	PCR–RFLP	14	42	44	15	43	76
	Selvihan Beysel	Caucasian	2019	HBC	PCR–RFLP	45	53	63	36	57	52
	Beibei Zhu	Asian	2019	PBC	iMLDR	0	240	34	0	353	27
FokI (rs2228570)						TT	CC	TC	TT	CC	TC
	Hesham A	African	2015	PBC	PCR–RFLP	34	24	54	65	33	120
	Mahmut Apaydın	Caucasian	2019	HBC	PCR–RFLP	16	41	43	8	80	46
	Selvihan Beysel	Caucasian	2019	HBC	PCR–RFLP	40	76	44	24	78	43
ApaI (rs7975232)						CC	AA	CA	CC	AA	CA
	Hesham A	African	2016	HBC	PCR–RFLP	25	51	81	9	55	93
	Mahmut Apaydın	Caucasian	2019	HBC	PCR–RFLP	31	17	52	32	26	76
	Selvihan Beysel	Caucasian	2019	HBC	PCR–RFLP	34	48	78	20	52	73
TaqI (rs731236)						CC	TT	CT	CC	TT	CT
	Golzar Rahmannezhad	Asian	2016	HBC	PCR–RFLP	16	78	63	17	55	85
	Mahmut Apaydın	Caucasian	2019	HBC	PCR–RFLP	14	44	42	14	54	66
	Selvihan Beysel	Caucasian	2019	HBC	PCR–RFLP	42	81	37	30	82	33
	Beibei Zhu	Asian	2019	PBC	iMLDR	8	237	29	21	341	18

PCR—RFLP, polymerase chain reaction—restriction fragment length polymorphism; iMLDR, improved multiple ligase detection reaction; HBC, hospital-based study; PBC, population-based study

### Publication bias

**Fig. 2:**
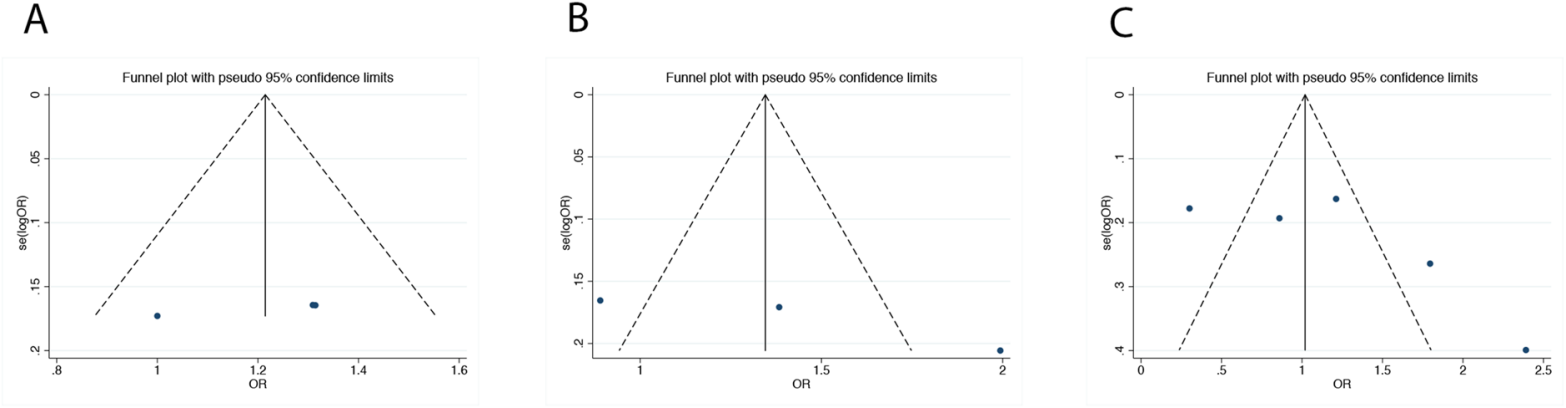
The funnel plot compared with the allele model for **a** ApaI (C vs A), **b** FokI (T vs C) and **c** BsmI (G vs A) gene polymorphisms to show publication bias

Funnel plot for comparison of allele models for ApaI (Fig. 2A), FokI (Fig. 2B) and BsmI (Fig. 2C) gene polymorphisms was evaluated to intuitively show the situation of publication bias. We used Begg’s test and Egger’s test to assess publication bias (Table 2). The results of the Egger’s test are P = 0.03 for the contrast of CT vs TT + CC of FokI (rs2228570), while the Begg’s test are P = 0.296. Publication bias was not observed in any other analysis under various other comparative models.

**Table 2:** Summary ORs (95% CI) of VDR gene polymorphisms and gestational diabetes mellitus risk

Site	Genetic model	Subgroup	Number	OR (95% CI)	P	P (Q test)	Egger	Begg
BsmI (rs1544410)	G vs A	Total	5	1.024 (0.512–2.048)	0.947	< 0.001	0.399	0.806
		Asian	2	1.959 (1.272–3.017)	**0.002**	0.549		
		African	1	0.301 (0.213–0.427)	** < 0.001**	1.000		
		Caucasian	2	1.037 (0.742–1.450)	0.832	0.174		
	GG vs AA	Total	3	0.523 (0.109–2.504)	0.417	< 0.001	0.616	1.000
		African	1	0.109 (0.051–0.232)	** < 0.001**	1.000		
		Caucasian	2	1.206 (0.749–1.941)	0.440	0.512		
	GA vs AA	Total	5	0.958 (0.414–2.214)	0.920	< 0.001	0.529	1.000
		Asian	2	2.059 (1.317–3.217)	**0.002**	0.472		
		African	1	0.234 (0.137–0.401)	** < 0.001**	1.000		
		Caucasian	2	0.885 (0.409–1.915)	0.757	0.045		
	GA + GG vs AA	Total	5	0.937 (0.378–2.323)	0.888	< 0.001	0.503	0.806
		Asian	2	2.059 (1.317–3.217)	**0.002**	0.472		
		African	1	0.188 (0.113–0.312)	** < 0.001**	1.000		
		Caucasian	2	0.940 (0.472–1.874)	0.861	0.053		
	GG vs AA + GA	Total	3	0.727 (0.261–2.025)	0.542	0.001		
		African	1	0.251 (0.126–0.498)	** < 0.001**	1.000		
		Caucasian	2	1.208 (0.789–1.852)	0.385	0.842		
	GA vs AA + GG	Total	5	1.075 (0.612–1.891)	0.800	< 0.001	0.358	0.462
		Asian	2	2.059 (1.317–3.217)	**0.002**	0.472		
		African	1	0.526 (0.329–0.840)	**0.007**	1.000		
		Caucasian	2	0.840 (0.444–1.589)	0.592	0.068		
FokI (rs2228570)	T vs C	Total	3	1.333 (0.852–2.085)	0.209	0.008	0.276	0.296
		African	1	0.890 (0.643–1.231)	0.481	1.000		
		Caucasian	2	1.631 (1.142–2.329)	**0.007**	0.172		
	TT vs CC	Total	3	1.612 (0.672–3.865)	0.285	0.012	0.597	1.000
		African	1	0.719 (0.368–1.405)	0.335	1.000		
		Caucasian	2	2.385 (1.079–5.272)	**0.032**	0.143		
	CT vs CC	Total	3	1.069 (0.593–1.929)	0.823	0.039	0.637	1.000
		African	1	0.619 (0.334–1.146)	0.127	1.000		
		Caucasian	2	1.372 (0.799–2.355)	0.252	0.159		
	CT + TT vs CC	Total	3	1.229 (0.659–2.293)	0.516	0.013		
		African	1	0.654 (0.365–1.173)	0.154	1.000		
		Caucasian	2	1.624 (0.992–2.661)	0.054	0.154		
	TT vs CC + CT	Total	3	1.454 (1.037–2.040)	**0.030**	0.096	0.281	0.296
		African	1	1.026 (0.625–1.686)	0.919	1.000		
		Caucasian	2	1.988 (1.235–3.200)	**0.005**	0.282		
	CT vs TT + CC	Total	3	0.964 (0.726–1.281)	0.803	0.191	0.03	0.296
		African	1	0.760 (0.482–1.200)	0.240	1.000		
		Caucasian	2	1.121 (0.780–1.611)	0.538	0.204		
Apal (rs7975232)	C vs A	Total	3	1.205 (0.998–1.456)	0.053	0.428	0.325	0.296
		Asian	1	1.309 (0.949–1.807)	0.101	1.000		
		Caucasian	2	1.154 (0.914–1.458)	0.228	0.252		
	CC vs AA	Total	3	1.974 (1.276–3.054)	**0.002**	0.479	0.816	1.000
		Asian	1	2.996 (1.278–7.022)	**0.012**	1.000		
		Caucasian	2	1.679 (1.006–2.804)	**0.048**	0.681		
	CA vs AA	Total	3	1.040 (0.760–1.422)	0.808	0.842	0.326	0.296
		Asian	1	0.939 (0.579–1.523)	0.800	1.000		
		Caucasian	2	1.119 (0.741–1.688)	0.593	0.820		
	CA + CC vs AA	Total	3	1.267 (0.940–1.708)	0.121	0.747	0.359	0.296
		Asian	1	1.121 (0.702–1.790)	0.633	1.000		
		Caucasian	2	1.378 (0.934–2.033)	0.106	0.704		
	CC vs AA + CA	Total	3	1.548 (1.080–2.217)	**0.017**	0.059	0.268	0.296
		Asian	1	3.114 (1.403–6.912)	**0.005**	1.000		
		Caucasian	2	1.258 (0.835–1.895)	0.272	0.189		
	CA vs AA + CC	Total	3	0.828 (0.632–1.085)	0.172	0.748	0.331	0.296
		Asian	1	0.733 (0.469–1.146)	0.174	1.000		
		Caucasian	2	0.889 (0.633–1.249)	0.497	0.719		
TaqI (rs731236)	C vs T	Total	4	0.985 (0.758–1.279)	0.907	0.099	0.519	0.734
		Asian	2	0.846 (0.582–1.231)	0.382	0.149		
		Caucasian	2	1.153 (0.896–1.484)	0.268	0.324		
	CC vs TT	Total	4	0.969 (0.681–1.379)	0.862	0.186	0.945	0.734
		Asian	2	0.605 (0.345–1.060)	0.079	0.740		
		Caucasian	2	1.356 (0.851–2.161)	0.200	0.780		
	CT vs TT	Total	4	1.000 (0.541–1.848)	0.999	0.002	0.465	1.000
		Asian	2	1.087 (0.252–4.677)	0.911	< 0.001		
		Caucasian	2	0.940 (0.633–1.394)	0.757	0.353		
	CT + CC vs TT	Total	4	0.949 (0.619–1.454)	0.810	0.022	0.199	0.734
		Asian	2	0.860 (0.351–2.112)	0.743	0.006		
		Caucasian	2	1.069 (0.731–1.563)	0.729	0.269		
	CC vs TT + CT	Total	4	1.049 (0.749–1.470)	0.781	0.225	0.963	1.000
		Asian	2	0.713 (0.417–1.219)	0.216	0.286		
		Caucasian	2	1.374 (0.882–2.139)	0.160	0.963		
	CT vs TT + CC	Total	4	0.984 (0.552–1.753)	0.956	0.002	0.575	1.000
		Asian	2	1.145 (0.281–4.663)	0.851	< 0.001		
		Caucasian	2	0.869 (0.599–1.263)	0.463	0.411		

OR, odds ratio; CI, confidence interval; vs, versus; P (Q test), P value of Q test for heterogeneity test; Bolded terms reflected P < 0.05

### ApaI (rs7975232)

**Fig. 3:**
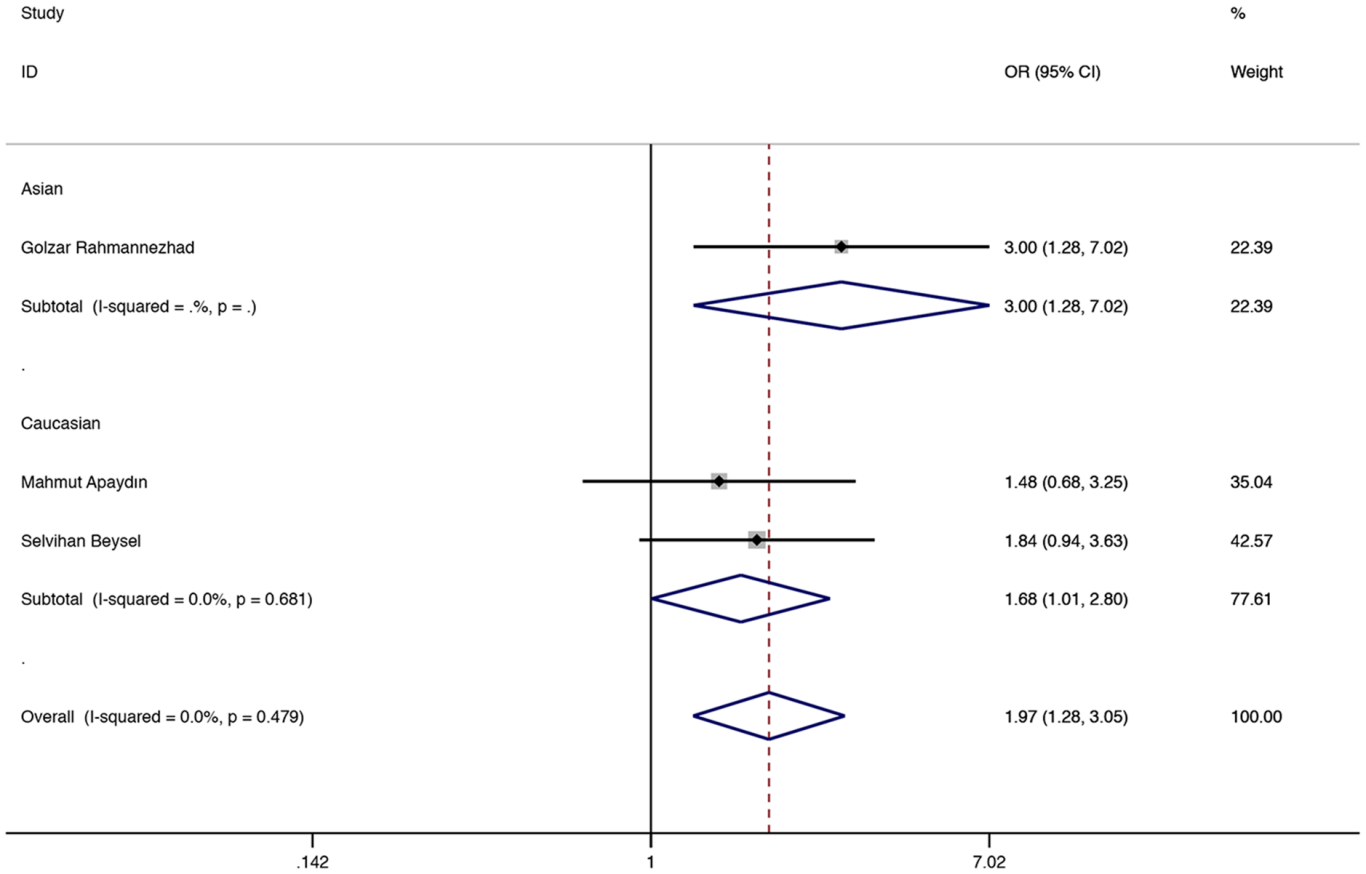
Fixed-effects meta-analysis on GDM risk and VDR ApaI (rs7975232) polymorphism in overall, Asian and Caucasian population (CC versus AA)

**Fig. 4:**
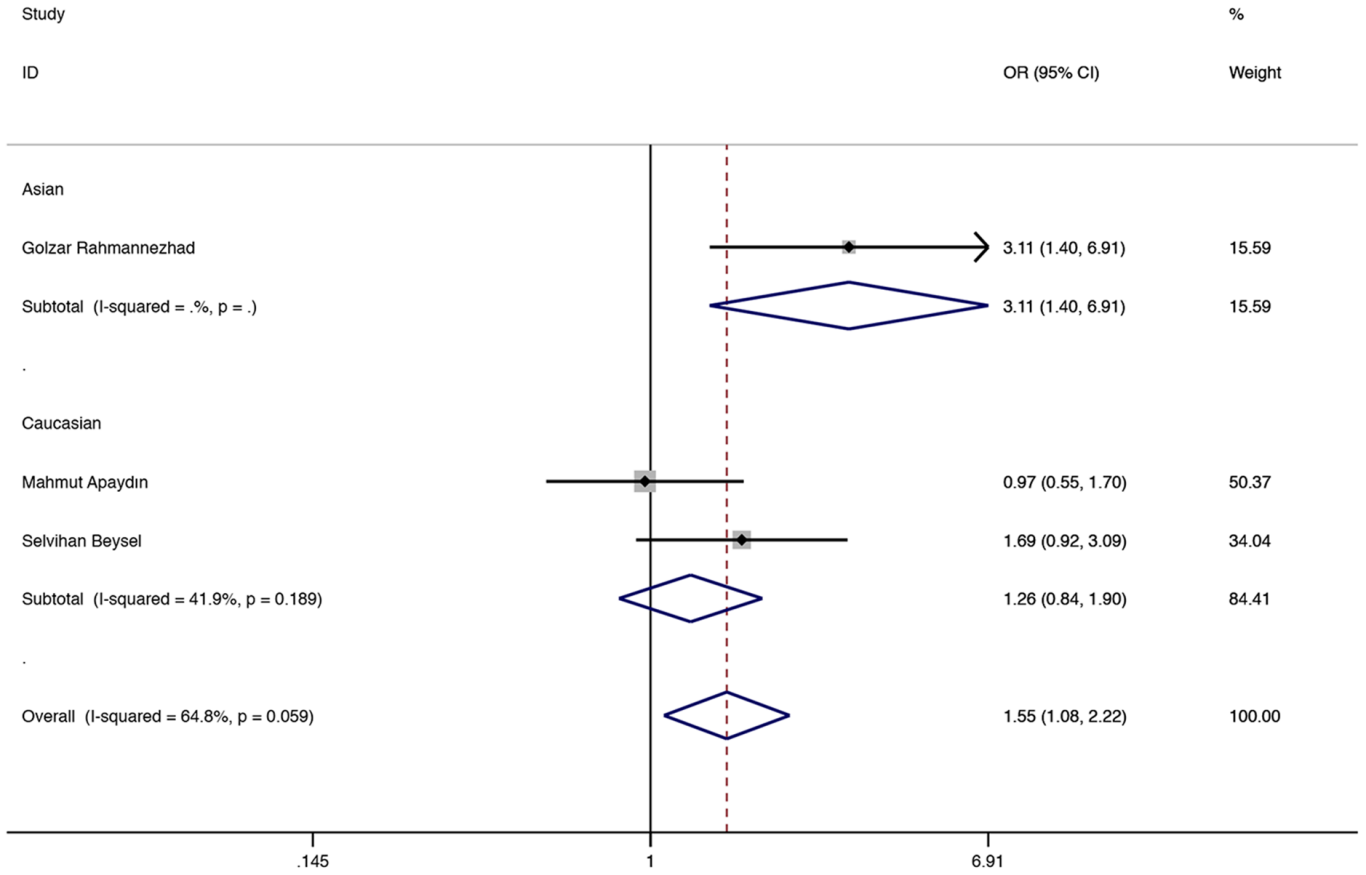
Fixed-effects meta-analysis on GDM risk and VDR ApaI (rs7975232) polymorphism under recessive model in overall and Asian population (CC vs AA + CA)

The results showed that in the total population of CC vs AA and the recessive model, ApaI (rs7975232) was associated with a higher GDM risk (CC vs AA: OR = 1.974, 95% CI 1.276–3.054, P = 0.002, Fig. 3; CC vs AA + CA: OR = 1.548, 95% CI 1.080–2.217, P = 0.017, Fig. 4). In the subgroup analysis, compared with the CC vs AA and recessive models in the Asian population, it was found to be associated with a higher risk of GDM (CC vs AA: OR = 2.996, 95% CI 1.278–7.022, P = 0.012, Fig. 3; CC vs AA + CA: OR = 3.114, 95% CI 1.403–6.912, P = 0.005, Fig. 4), and CC vs AA comparison among Caucasian populations. (CC vs AA: OR = 1.679, 95% CI 1.006–2.804, P = 0.048, Fig. 3). Table 2 shows other related results of ApaI (rs7975232).

### FokI (rs2228570)

**Fig. 5:**
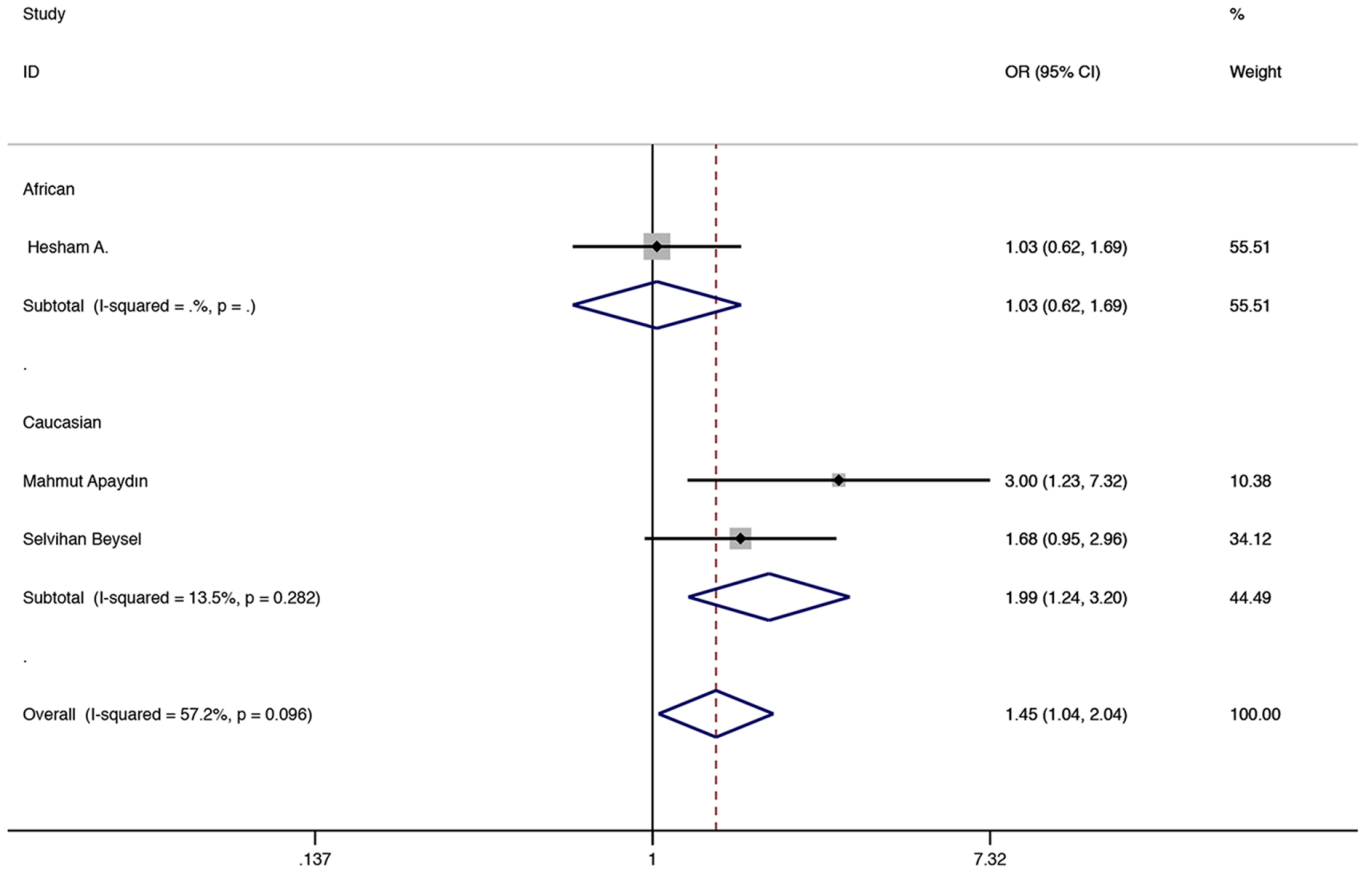
Fixed-effects meta-analysis on GDM risk and VDR FokI (rs2228570) polymorphism under recessive model in overall and Caucasian population (TT vs CC + CT)

**Fig. 6:**
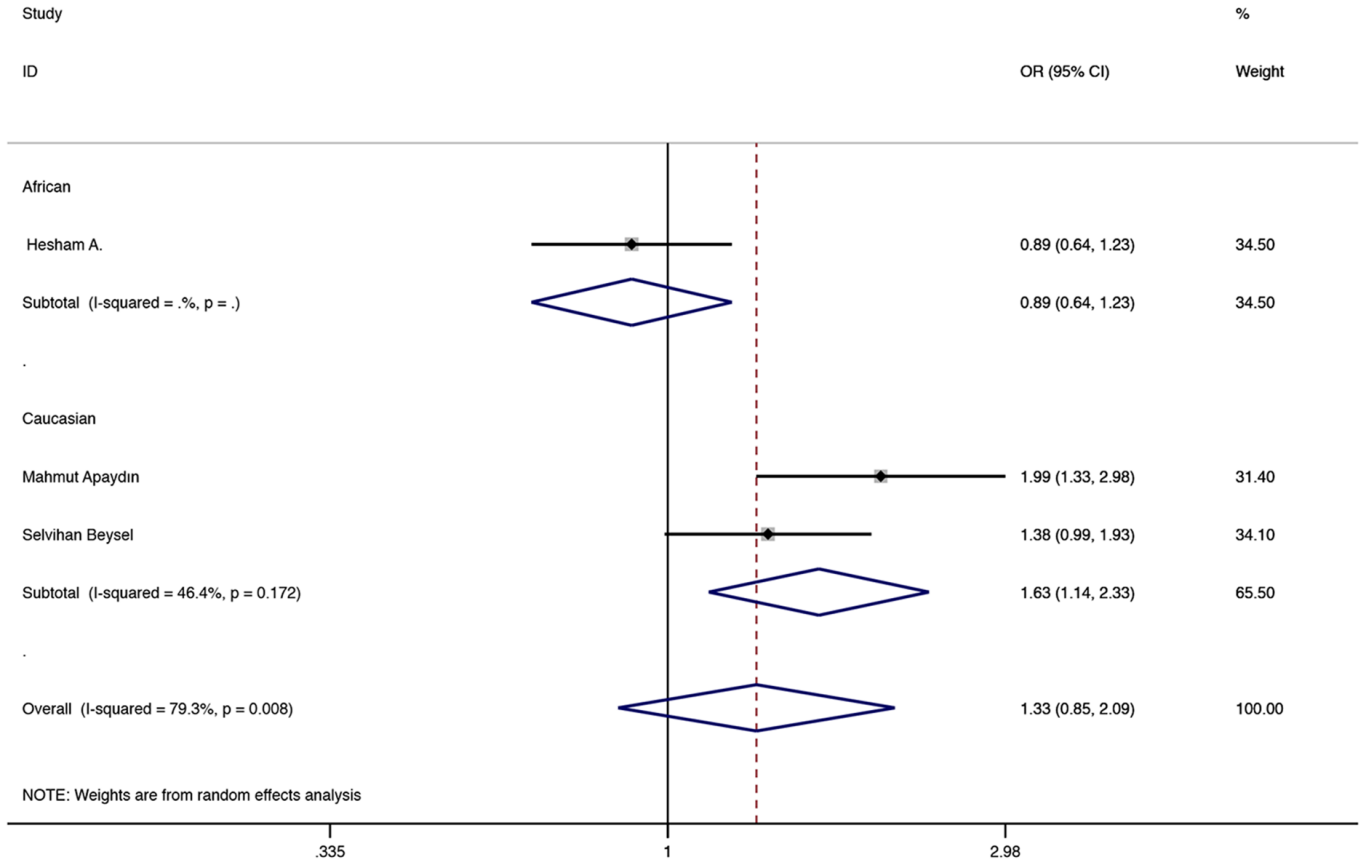
Random-effects meta-analysis on GDM risk and VDR FokI (rs2228570) polymorphism under allelic model in Caucasian population (T vs C)

The results showed that in the recessive model, FokI (rs2228570) was associated with a higher GDM risk in overall population (TT vs CC + CT: OR = 1.454, 95% CI 1.037–2.040, P = 0.030, Fig. 5). In the subgroup, a relationship with a higher GDM risk was found in the Caucasian population under the allele and recessive models (T vs C: OR = 1.631, 95% CI 1.142–2.329, P = 0.007, Fig. 6; TT vs CC + CT: OR = 1.988, 95% CI 1.235–3.200, P = 0.005, Fig. 5). The other related results of FokI (rs2228570) were shown in Table 2.

### BsmI (rs1544410)

**Fig. 7:**
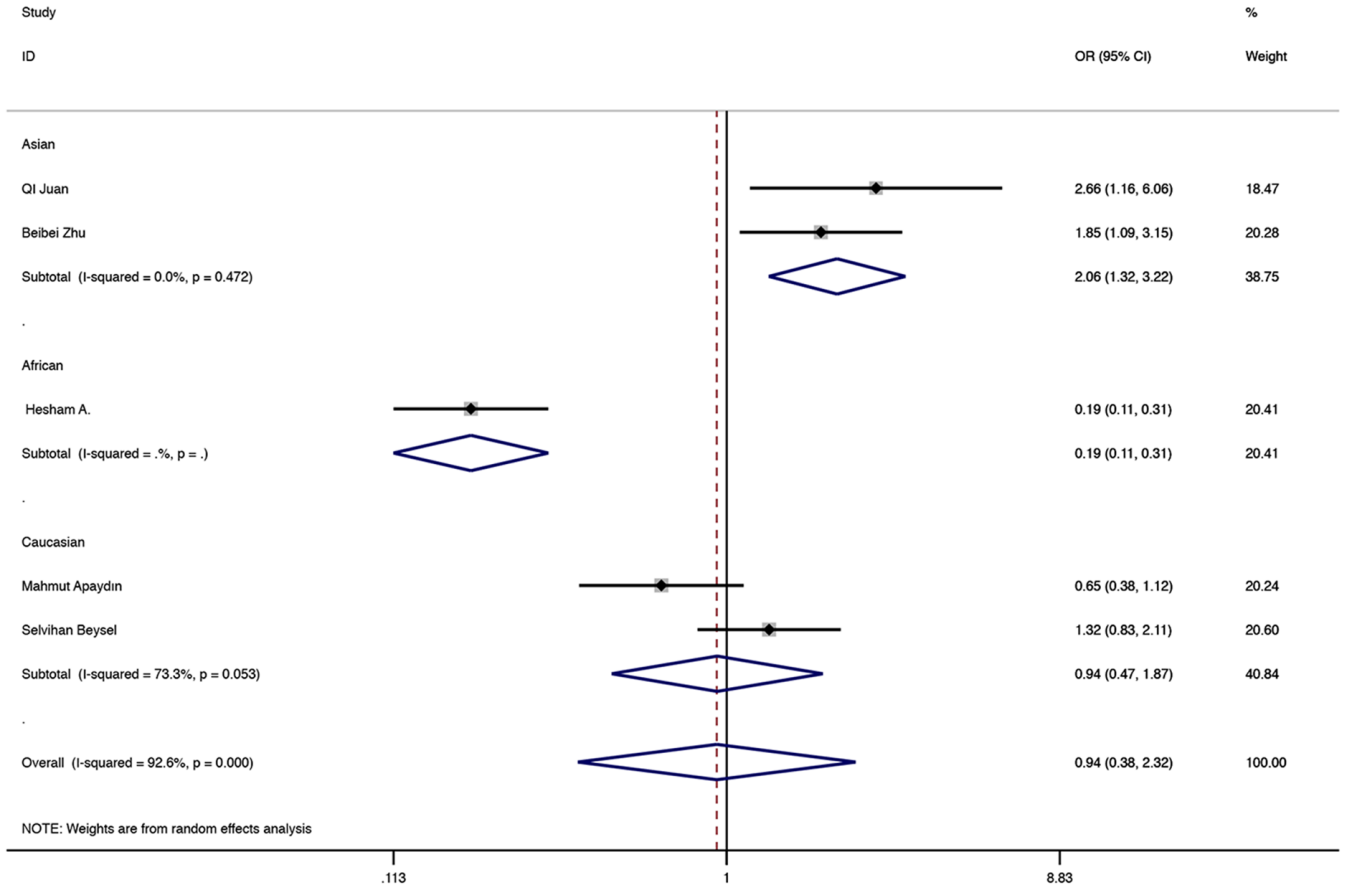
Random-effects meta-analysis on GDM risk and VDR BsmI (rs1544410) polymorphism under dominant model in Asian and African population (GA + GG vs AA)

**Fig. 8:**
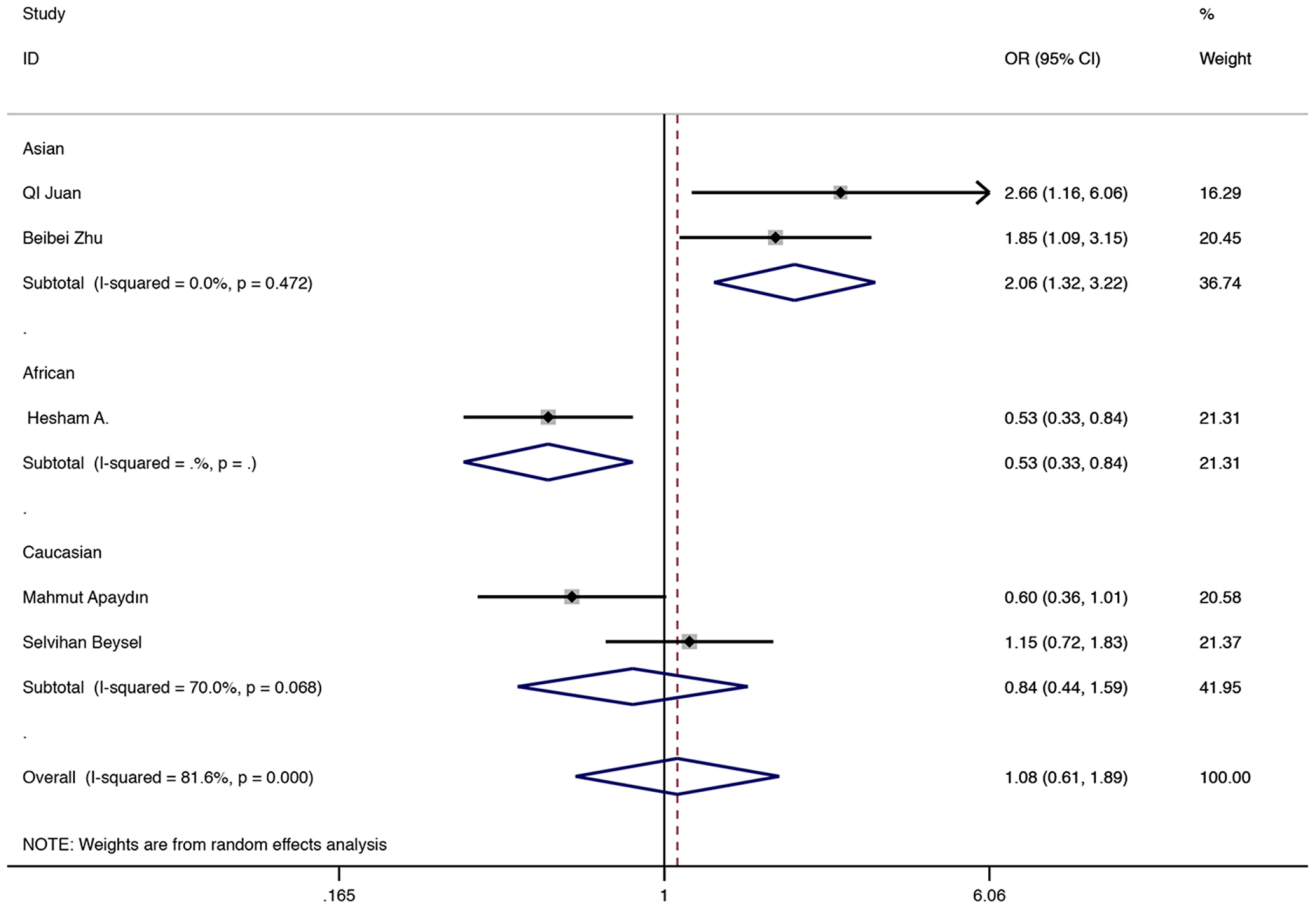
Random-effects meta-analysis on GDM risk and VDR BsmI (rs1544410) polymorphism under over-dominant model in Asian and African population (GA vs AA + GG)

The results showed that BsmI (rs1544410) was not related to GDM risk in the general population. In the subgroup, a relationship with higher GDM risk was found in the Asian population allele model, the comparison of GA vs AA, the dominant model and the over-dominant model. (G vs A: OR = 1.959, 95% CI 1.272–3.017, P = 0.002; GA vs AA: OR = 2.059, 95% CI 1.317–3.217, P = 0.002; GA + GG vs AA: OR = 2.059 95% CI 1.317–3.217, P = 0.002, Fig. 7; GA versus AA + GG: OR = 2.059, 95% CI 1.317–3.217, P = 0.002, Fig. 8). In the subgroup, relationships with lower GDM risk were found in African populations through allele models, GG vs AA, GA vs AA, dominant, recessive and over-dominant models (G vs A: OR = 0.301, 95% CI 0.213–0.427, P < 0.001; GG vs AA: OR = 0.109, 95% CI 0.051–0.232, P < 0.001; GA vs AA: OR = 0.234, 95% CI 0.137–0.401, P < 0.001; GA + GG vs AA: OR = 0.188, 95% CI 0.113–0.312, P < 0.001, Fig. 7; GG vs AA + GA: OR = 0.251, 95% CI 0.126–0.498, P < 0.001; GA vs AA + GG: OR = 0.526, 95% CI 0.329–0.840, P = 0.007, Fig. 8). Other related results of BsmI (rs1544410) were shown in Table 2.

### TaqI (rs731236)

The data showed that the TaqI (rs731236) polymorphism of the VDR gene was not related to susceptibility to GDM (Table 2). TaqI (rs731236) was heterogeneous in CT and TT contrast, overt dominant models, and overdominant models in overall population. In the subgroup, CT versus TT showed heterogeneity between the dominant model and the over-dominant model (Table 2).

### Sensitivity analyses

One-way sensitivity analysis was performed on the data involved in this meta-analysis. Each study of the meta-analysis was deleted to reflect the overall impact of each data set, and the corresponding combined results did not change substantially.

## Discussion

GDM has become major health concern worldwide. Studies suggested that VDR gene polymorphisms might have an impact on GDM risk [14, 16, 18]. However, it is difficult to obtain more accurate results through a single study to determine the relationship between genes and diseases. Meta-analysis can solve the problem of insufficient statistics in a single study, so as to draw more precise conclusions. The association of VDR gene polymorphisms with the incidence of cancer, osteoporosis, and autoimmune thyroid disease has been confirmed in a meta-analysis [20–22]. In our study the PICO was shown as follow: P: Gestational diabetes mellitus; I: vitamin D receptor (VDR) polymorphisms; C: control people; O: susceptibility. This study showed ApaI (rs7975232) VDR gene polymorphism was related with GDM for the comparison of CC vs AA and recessive model in overall population and FokI (rs2228570) VDR gene polymorphism was associated with the risk of GDM for recessive model in overall population. The BsmI (rs1544410) and TaqI (rs731236) polymorphisms of the VDR were not related with GDM in overall population.

Due to differences between races, evidence that could cause disease is sometimes not very reliable. This suggests that different races influence genetic background differently [23]. Therefore, based on subgroup analysis of different races, it can be found that the same polymorphisms in disease susceptibility in different populations play different roles. In our study, subgroup analysis suggested that the VDR gene ApaI (rs7975232) polymorphism was significantly associated with GDM for the comparison of CC vs AA and recessive model in Asian population and under the comparison of CC vs AA in Caucasian population. For VDR gene FokI (rs2228570) polymorphism, it was significantly associated with GDM under the comparison of CC vs AA and the recessive model in Asian and under allelic model and the recessive model in Caucasian. However, for VDR gene BsmI (rs1544410) polymorphism, it was significantly associated with GDM under allelic model, the comparison of GA vs AA, dominant model, and over-dominant model in Asian and under allelic model, the comparison of GG vs AA, the comparison of GA vs AA, dominant model, recessive model and over-dominant model in African population. Interestingly, the subgroup analysis in Asia and Africa for BsmI (rs1544410) is the opposite, perhaps because of ethnic differences. Of course, it also may be the difference in results caused by the insufficient number of studies included. We certainly need more and better research to get more reliable results.

However, this meta-analysis has some limitations. Firstly, heterogeneity may influence the results of this meta-analysis. Nonetheless, we use specific research standards to strictly perform data extraction and analysis to minimize this possibility. Secondly, the study only includes published studies, and the existence of results indicating no meaning or negative results may not be published, and this will increase the likelihood of publication bias. Finally, our results have not been adjusted. If you can get more research data, you should be able to analyze it more accurately. We can obtain more accurate results by adjusting other variables, including age and family history, etc. [24–27]. In addition, an in-depth analysis of these factors provides a more complete understanding of the linkages between these factors and the risks of GDM.

## Conclusions

In summary, VDR ApaI (rs7975232) and FokI (rs2228570) polymorphisms increase susceptibility to GDM. In the future, it can be used to diagnose and screen molecular biomarkers for GDM patients. VDR BsmI (rs1544410) polymorphism was associated with GDM in Asian and African population. VDR TaqI (rs731236) polymorphism was not associated with GDM.

## Data Availability

The datasets used and analyzed in the present study are available from the corresponding author upon reasonable request.

## Abbreviations

GDM: Gestational diabetes mellitus
VDR: Vitamin D receptor
OR: Odds ratio
CI: Confidence interval

## References

[1] Zhang C, Bao W, Rong Y, Yang H, Bowers K, Yeung E, Kiely M (2013). Genetic variants and the risk of gestational diabetes mellitus: a systematic review. Hum Reprod Update.

[2] Mao H, Li Q, Gao S (2012). Meta-analysis of the relationship between common type 2 diabetes risk gene variants with gestational diabetes mellitus. PloS ONE.

[3] Huopio H, Cederberg H, Vangipurapu J, Hakkarainen H, Paakkonen M, Kuulasmaa T, Heinonen S, Laakso M (2013). Association of risk variants for type 2 diabetes and hyperglycemia with gestational diabetes. Eur J Endocrinol.

[4] Lauenborg J, Grarup N, Damm P, Borch-Johnsen K, Jorgensen T, Pedersen O, Hansen T (2009). Common type 2 diabetes risk gene variants associate with gestational diabetes. J Clin Endocrinol Metab.

[5] Schneider S, Bock C, Wetzel M, Maul H, Loerbroks A (2012). The prevalence of gestational diabetes in advanced economies. J Perinat Med.

[6] Haussler MR, Whitfield GK, Haussler CA, Hsieh JC, Thompson PD, Selznick SH, Dominguez CE, Jurutka PW (1998). The nuclear vitamin D receptor: biological and molecular regulatory properties revealed. J Bone Miner Res.

[7] Uitterlinden AG, Fang Y, Van Meurs JB, Pols HA, Van Leeuwen JP (2004). Genetics and biology of vitamin D receptor polymorphisms. Gene.

[8] Uitterlinden AG, Pols HA, Burger H, Huang Q, Van Daele PL, Van Duijn CM, Hofman A, Birkenhager JC, Van Leeuwen JP (1996). A large-scale population-based study of the association of vitamin D receptor gene polymorphisms with bone mineral density. J Bone Miner Res.

[9] Palomer X, Gonzalez-Clemente JM, Blanco-Vaca F, Mauricio D (2008). Role of vitamin D in the pathogenesis of type 2 diabetes mellitus. Diabetes Obes Metab.

[10] Ban Y, Taniyama M, Yanagawa T, Yamada S, Maruyama T, Kasuga A, Ban Y (2001). Vitamin D receptor initiation codon polymorphism influences genetic susceptibility to type 1 diabetes mellitus in the Japanese population. BMC Med Genet.

[11] Skrabic V, Zemunik T, Situm M, Terzic J (2003). Vitamin D receptor polymorphism and susceptibility to type 1 diabetes in the Dalmatian population. Diabetes Res Clin Pract.

[12] Liu Z, Liu L, Chen X, He W, Yu X (2014). Associations study of vitamin D receptor gene polymorphisms with diabetic microvascular complications: a meta-analysis. Gene.

[13] Rahmannezhad G, Mashayekhi FJ, Goodarzi MT, Rezvanfar MR, Sadeghi A (2016). Association between vitamin D receptor ApaI and TaqI gene polymorphisms and gestational diabetes mellitus in an Iranian pregnant women population. Gene.

[14] Apaydin M, Beysel S, Eyerci N, Pinarli FA, Ulubay M, Kizilgul M, Ozdemir O, Caliskan M, Cakal E (2019). The VDR gene FokI polymorphism is associated with gestational diabetes mellitus in Turkish women. BMC Med Genet.

[15] Beysel S, Eyerci N, Ulubay M, Caliskan M, Kizilgul M, Hafizoglu M, Cakal E (2019). Maternal genetic contribution to pre-pregnancy obesity, gestational weight gain, and gestational diabetes mellitus. Diabetol Metab Syndr.

[16] Zhu B, Huang K, Yan S, Hao J, Zhu P, Chen Y, Ye A, Tao F (2019). VDR variants rather than early pregnancy vitamin D concentrations are associated with the risk of gestational diabetes: the Ma'anshan Birth Cohort (MABC) study. J Diabetes Res.

[17] El-Beshbishy HA, Tawfeek MA, Taha IM, FadulElahi T, Shaheen AY, Bardi FA, Sultan II (2015). Association of vitamin D receptor gene BsmI (A>G) and FokI (C>T) polymorphism in gestational diabetes among Saudi women. Pak J Med Sci.

[18] Siqueira TW, Araujo Junior E, Mattar R, Daher S (2019). Assessment of polymorphism of the VDR gene and serum vitamin D values in gestational diabetes mellitus. Revista Brasileira de Ginecologia e Obstetricia.

[19] Qi J, Xu Y (2013). Single nucleotide polymorphism of vitamin D receptor gene in patient with gestational diabetes mellitus. Lab Med Clin.

[20] Rai V, Abdo J, Agrawal S, Agrawal DK (2017). Vitamin D receptor polymorphism and cancer: an update. Anticancer Res.

[21] Zhang L, Yin X, Wang J, Xu D, Wang Y, Yang J, Tao Y, Zhang S, Feng X, Yan C (2018). Associations between VDR gene polymorphisms and osteoporosis risk and bone mineral density in postmenopausal women: a systematic review and meta-analysis. Sci Rep.

[22] Gao XR, Yu YG (2018). Meta-analysis of the association between vitamin D receptor polymorphisms and the risk of autoimmune thyroid disease. Int J Endocrinol.

[23] Hirschhorn JN, Lohmueller K, Byrne E, Hirschhorn K (2002). A comprehensive review of genetic association studies. Genet Med.

[24] Chiefari E, Arcidiacono B, Foti D, Brunetti A (2017). Gestational diabetes mellitus: an updated overview. J Endocrinol Invest.

[25] Garrison A (2015). Screening, diagnosis, and management of gestational diabetes mellitus. Am Fam Physician.

[26] Coustan DR (2013). Gestational diabetes mellitus. Clin Chem.

[27] Ashwal E, Hod M (2015). Gestational diabetes mellitus: where are we now?. Clinica Chimica Acta.

